# Three new species of mygalomorph and filistatid spiders from Iran (Araneae, Cyrtaucheniidae, Nemesiidae and Filistatidae)

**DOI:** 10.3897/zookeys.463.8692

**Published:** 2014-12-12

**Authors:** Yuri M. Marusik, Alireza Zamani, Omid Mirshamsi

**Affiliations:** 1 Institute for Biological Problems of the North RAS, Portovaya Str. 18, Magadan 685000, Russia; 2 Zoological Museum, University of Turku, FI-20014 Turku, Finland; 3 Department of Animal Biology, School of Biology and Center of Excellence in Phylogeny of Living Organisms, College of Science, University of Tehran, Tehran, Iran; 4 Department of Biology, Faculty of Sciences, Ferdowsi University of Mashhad, Mashhad, Iran; 5 Institute of Applied Zoology, Faculty of Sciences, Ferdowsi University of Mashhad, Mashhad, Iran

**Keywords:** *Anemesia*, *Raveniola*, *Sahastata*, type species, new record

## Abstract

Three new spider species are described from Iran: *Anemesia koponeni* **sp. n.** (♂, Cyrtaucheniidae); *Raveniola mazandaranica* **sp. n.** (♂, Nemesiidae) and *Sahastata sinuspersica* **sp. n.** (♀, Filistatidae). Cyrtaucheniidae and *Sahastata* Benoit, 1968 are reported from Iran for the fisrt time.

## Introduction

Spiders of Iran remain poorly studied in faunistic and especially taxonomic respects. Although about 540 species are known from the country (Zamani et al. 2014a), this number is smaller than that from the neighboring and much smaller Azerbaijan (714 species, Otto 2014). There are many small faunistic publications dealing usually with common spiders (e.g. Mirshamsi 2005, Ghahari and Marusik 2009, Ghahari and Tabari 2012, Kashefi et al. 2013), but taxonomic and large scale faunistic surveys are almost lacking (Mozaffarian and Marusik 2001, Logunov et al. 2001, Ono and Martens 2005, Ghavami 2006, Moradmand and Jäger 2011, Zamani et al. 2014b).

While studying spiders of Iran, we found three undescribed species belonging to Mygalomorphae and Filistatidae, two of which represent the first Iranian records of the family Cyrtaucheniidae and the genus *Sahastata* Benoit, 1968 (Filistatidae).

## Material and methods

Photographs were taken in dishes of different sizes with paraffin at the bottom. Specimens were photographed using an Olympus Camedia E-520 camera attached to an Olympus SZX16 stereomicroscope at the Zoological Museum, University of Turku. Digital images were prepared using “CombineZP” image stacking software. Illustrations of endogynes were made after maceration in 20% potassium hydroxide aqueous solution and exposition for few minutes in alcohol/water solution of Chlorazol Black. Lengths of leg segments were measured on the dorsal side. All measurements are given in mm. Treated materials will be deposited in Senckenberg Museum, Frankfurt am Main (SMF).

List of abbreviations is as follows: AME, anterior median eyes; ALE, anterior lateral eyes; PME, posterior median eyes; PLE, posterior lateral eyes; PMS, posterior median spinnerets; PLS, posterior lateral spinnerets. For describing the spination patterns, the following abbreviations or their combinations are used: a, apical; d, dorsal; m, median; p, prolateral; r, retrolateral; v, ventral.

## Taxonomic survey

### Cyrtaucheniidae Simon, 1892

#### Genus Anemesia

Taxon classificationAnimaliaAraneaeCyrtaucheniidae

Pocock, 1895

##### Comments.

*Anemesia* is a small genus of Cyrtaucheniidae with four species known from Afghanistan, Tajikistan, Turkmenistan and Uzbekistan (Platnick 2014). Species of this genus have been treated in six papers only (Pocock 1895, Spassky 1937, Andreeva 1968, 1976, Charitonov 1969, Zonstein 2001).

#### Anemesia koponeni sp. n.

Taxon classificationAnimaliaAraneaeCyrtaucheniidae

http://zoobank.org/EEDAEB57-B0E0-4CA9-A18F-6FEDF1DF9669

Figs 1–8

##### Material.

Holotype ♂ (SMF) – IRAN: ***Khorāsān-e Jonoubi*** Province, Qāen County, Kārizan (33°53'N, 59°49'E), May 1, 2012 (O. Mirshamsi).

##### Etymology.

Named after our colleague and friend Seppo Koponen (Turku, Finland), a famous Finnish arachnologist on occasion of his 70th birthday; noun.

##### Diagnosis.

The new species has a rather short embolus like in *Anemesia birulai* (Spassky, 1937) (cf. Spassky 1937, fig. 2). Other congeners occurring in Central Asia have distinctly longer emboli, for example *Anemesia karatauvi* (Andreeva, 1968) and *Anemesia incana* Zonstein, 2001 (Figs 9–10). *Anemesia koponeni* sp. n. differs from the former species by larger body size (15 *vs.* 10 mm), much darker general coloration (chestnut-brown *vs.* light yellowish-brown), as well as by longer and spinose palpal tibia (shorter and aspinose in *Anemesia birulai*). Finally it differs, from the similar *Anemesia tubifex* (Pocock, 1889) by its narrower eye field with the AME closer to each other (cf. Pocock 1889, fig. 2d).

**Figures 1–11.**
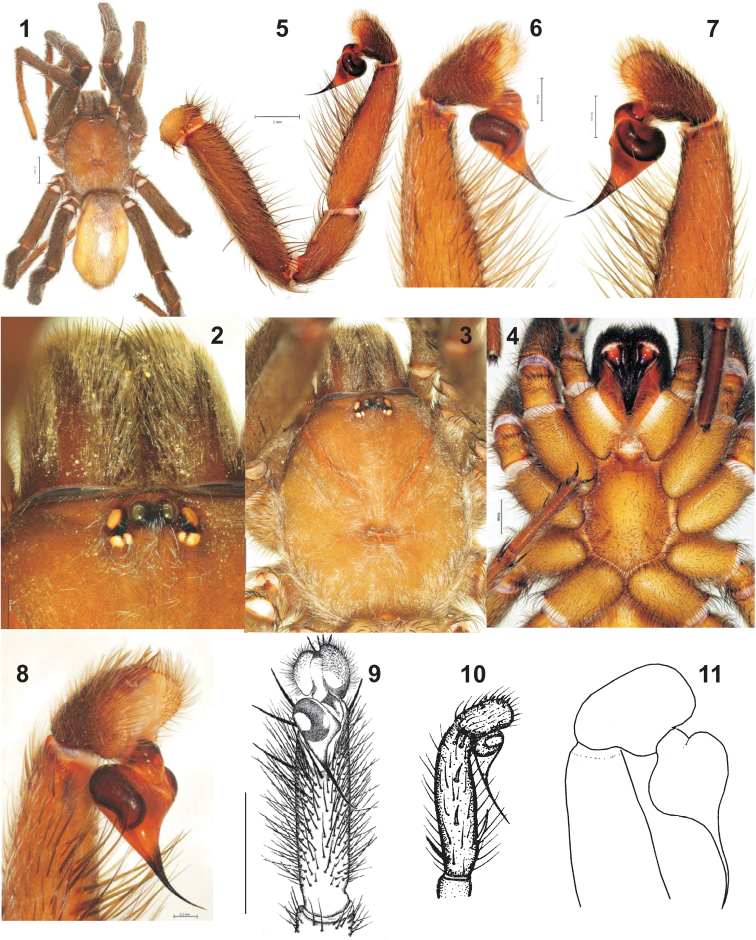
Habitus and male palp of four *Anemesia* species: *Anemesia koponeni* sp. n. (**1–8**), *Anemesia incana* (**9**, after Zonstein 2001), *Anemesia karatauvi* (**10**, after Andreeva 1976) and *Anemesia birulai* (**11**, after Spassky 1937, modified). **1** male habitus, dorsal **2** eye group and chelicerae, dorsal **3–4** prosoma, dorsal and ventral **5** whole palp, retrolateral **6–8** tip of palp, prolateral, retrolateral and prolateral-apical **9** palp, ventral **10–11** palp, prolateral.

##### Description.

Total length 15.2 including chelicerae. Color in alcohol: carapace, palps and most part of legs reddish-brown; eye tubercle brownish-black; clypeus, chelicerae, femora I–II dorsally dark reddish-brown; sternum, labium, maxillae, palps and legs ventrally yellowish-brown; abdomen dorsally with an indistinct pattern consisting of a short median stripe and a few pairs of interrupted transverse fasciae; metatarsi III-IV, tarsi I-IV, ventral abdominal surface and spinnerets light yellowish-brown. Carapace 6.0 long, 5.2 wide. Eye sizes and interspaces: AME 0.17, ALE 0.25, PLE 0.20, PME 0.12, AME–AME 0.20. Cheliceral rastellum weak. Maxillae with about 15 small cuspules each. Sternum 3.15 long, 2.50 wide.

Spination. Palp: femur 3d, 2pd; patella 1p; tibia 2v; tarsus 10d. Leg I: femur 4d, 3pd, 3rd; patella 2p; tibia 3p, 3r, 6-8v; metatarsus 1d, 1p, 1r, 5v. Leg II: femur 4d, 3pd, 3rd; patella 2p; tibia 3p, 2r, 9v; metatarsus 2d, 3p, 1r, 7v. Leg III: femur 3d, 3pd, 3rd; patella 2p, 1r; tibia 1d, 3p, 3r, 6v; metatarsus 3p, 4r, 7v; tarsus 2v. Leg IV: femur 3d, 3pd, 3rd; tibia 1d, 1p, 3r, 6v; metatarsus 1d, 2p, 5r, 7v; tarsus 2v. Patella IV and tarsi I–II aspinose.

Scopula: distal on metatarsi I–II, present on tarsi I-III, absent on tarsus IV. Paired claws: inner and outer margins with 6–7 teeth each. Spinnerets: PMS 0.53 long; PLS 2.25 long; apical segment triangle.

Palp as in Figs 5–8, thin, femur slightly longer than tibia, and as long as patella; bulb as long as patella, bulb (with embolus) 2.3 longer than widest diameter of bulb; embolus without distinct base, its length subequal to length of bulb.

##### Distribution.

The species is known only from the type locality. It is the southwesternmost record of the genus.

### Nemesiidae Simon, 1889

#### Genus Raveniola

Taxon classificationAnimaliaAraneaeNemesiidae

Zonstein, 1987

##### Comments.

*Raveniola* is a relatively large genus of nemesiid spiders with 22 described species. The genus is distributed from Turkey to China (cf. Platnick 2014). Two species of this genus are known from Iran: *Raveniola niedermeyeri* (Brignoli, 1972) and *Raveniola vonwicki* Zonstein, 2000. Both species are well studied by Zonstein (2000) and Zonstein and Marusik (2010).

#### Raveniola mazandaranica sp. n.

Taxon classificationAnimaliaAraneaeNemesiidae

http://zoobank.org/A43C76C0-5750-4B2C-9616-F747BA48DA06

Figs 12–18

##### Material.

Holotype ♂ (SMF), IRAN, ***Māzandarān*** Province, Abbās Abād, Salmān Shahr, around Dāniāl Cave (36°39'N, 51°10'E), April 2014 (P. Beyhaghi).

##### Etymology.

The specific epithet is derived from the type locality; adjective.

##### Diagnosis.

So far, only two *Raveniola* species are known to occur in Northern Iran: *Raveniola niedermeyeri* and *Raveniola vonwicki*. The new species can be distinguished from *Raveniola niedermeyeri* by its twisted embolus (gradually bent in *Raveniola niedermeyeri*, Fig. 21). *Raveniola mazandaranica* sp. n. differs from the similar *Raveniola vonwicki* by the shape of its embolic tip and by having a tibial prolateral apical spine (arrowed on Fig. 15), lacking in sibling species. All three Iranian species differ in the number of their prolateral apical tibial spines: none in *Raveniola vonwicki*, two in *Raveniola niedermeyeri*, and one in *Raveniola mazandaranica* sp. n.

**Figures 12–21.**
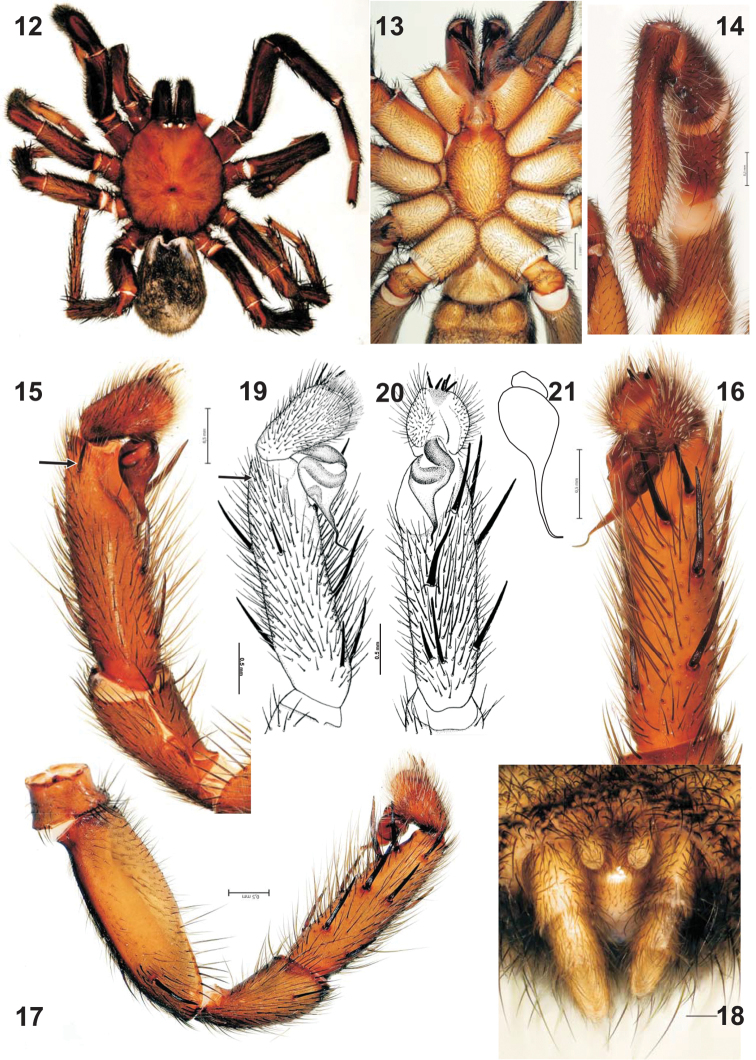
Habitus and male palp of three *Raveniola* species: *Raveniola mazandaranica* sp. n. (**12–18**), *Raveniola vonwicki* (**19–20**, after Zonstein 2000) and *Raveniola niedermeyeri* (**21**). **12** male habitus, dorsal **13** prosoma, ventral **14** leg I, ventral **15**, **19** palp, prolateral **16**, **20** palp, ventral **17** whole palp, retrolateral **18** spinnerets, ventral **21** bulb, ventral.

##### Description.

Total length 11.3 with chelicerae and 9.7 without chelicerae. Carapace 5.25 long, 4.9 wide; sternum 2.55 long, 1.85 wide; eyefield 1.13 wide, 0.55 long. Habitus and pattern as in Figs 12–13. Eye sizes and interspaces: AME 0.15, ALE 0.3, PLE 0.2, PME 0.17, AME–AME 0.12. Maxillae with about 10 cuspules each. Palp: 2.75 + 1.55 + 2.25 + 0.9, bulb (including embolus) 0.83 long. Leg I: 4.0 + 2.3 + 3.35 + 3.25 + 1.8. Leg II: 3.85 + 2.0 + 2.75 + 2.75 + 1.8. Leg III: 3.37 + 1.7 + 3.25 + 3.3 + 1.75. Leg IV: 4.2 + 2.0 + 3.35 + 4.75 + 2.0. Metatarsus I modified, with thinner proximal half and thicker distal half (Fig. 14). Spination of palp: femur 2d, 1pa; patella 0; tibia 2d, 1ra, 3p, 3vp, 2vr, 1vm; cymbium 4. Leg I: femur d4, p21; tibia p2, v6. Leg II: femur d4, p3; patella p1; tibia p3, v7; metatarsus p1, v6. Leg III: femur d4, p3, r2; patella p1, r1; tibia d2, p3, r3, v7; metatarsus d2, p3, r3, v8. Leg IV: femur d4, p3, r2; patella p2, r1; tibia d2, p4, r4, v7; metatarsus d5, p4, r4, v10. Scopula: present on metatarsi I–II and tarsi I–II; absent on tarsi III–IV. Paired claws: inner and outer margins with 7–11 teeth each. Spinnerets: PMS 0.3 long; PLS 1.2 long, apical segment triangle.

Palp as in Figs 15–17, femur slightly longer than tibia and about 1.5 thicker; patella longer than cymbium and wider than tibia; tibia prolaterally with one apical spine; bulb 2.7 shorter than tibia, embolus bent 3 times.

##### Distribution.

The new species is known only from the type locality.

### Filistatidae Simon, 1864

#### Sahastata

Taxon classificationAnimaliaAraneaeFilistatidae

Benoit, 1968

##### Comments.

*Sahastata* is a small genus of Filistatinae spiders with three described species. It is known from the Mediterranean to India (Platnick 2014). Members of this genus differ distinctly from *Filistata* by their very hairy sternum+labium and a calamistrum having 2–3 rows of inclined hairs.

#### Sahastata sinuspersica sp. n.

Taxon classificationAnimaliaAraneaeFilistatidae

http://zoobank.org/DE576BD3-D04B-486D-92C2-653FACA564EF

Figs 22–29 , 34–40

##### Material.

Holotype ♀ (SMF), IRAN, ***Hormozgān*** Province, Bandar-e‘Abbās (found under a large rock, in a sandy substrate near the harbor), 27°11'N, 56°17'E, January 2014 (A. Zamani). Paratypes: 1♀ and 3 juv (SMF), IRAN, ***Hormozgān*** Province, Hormuz Island (found under a large rock, in a sandy substrate near the sea), 27°04'N, 56°28'E, January 2014 (A. Zamani).

##### Comparative material.

Syntype ♀ of *Sahastata nigra* (Simon, 1897) from Muscat, in Muséum National d’Histoire Naturelle, Paris.

##### Etymology.

The specific epithet is derived from the Persian Gulf (Sinus Persicus in Latin); adjective.

##### Diagnosis.

The new species differs distinctly from the type species, *Sahastata nigra* by its lighter coloration and undivided receptacles (with two heads in *Sahastata nigra*, Fig. 41). It differs from *Sahastata sabaea* Brignoli, 1982 by its receptacles which are twice as large in size (6.1 in *Sahastata sabaea*) and unbranched (with two “heads” in *Sahastata sabaea*, f. 18, Brignoli 1982).

##### Description.

**Figures 22–33.**
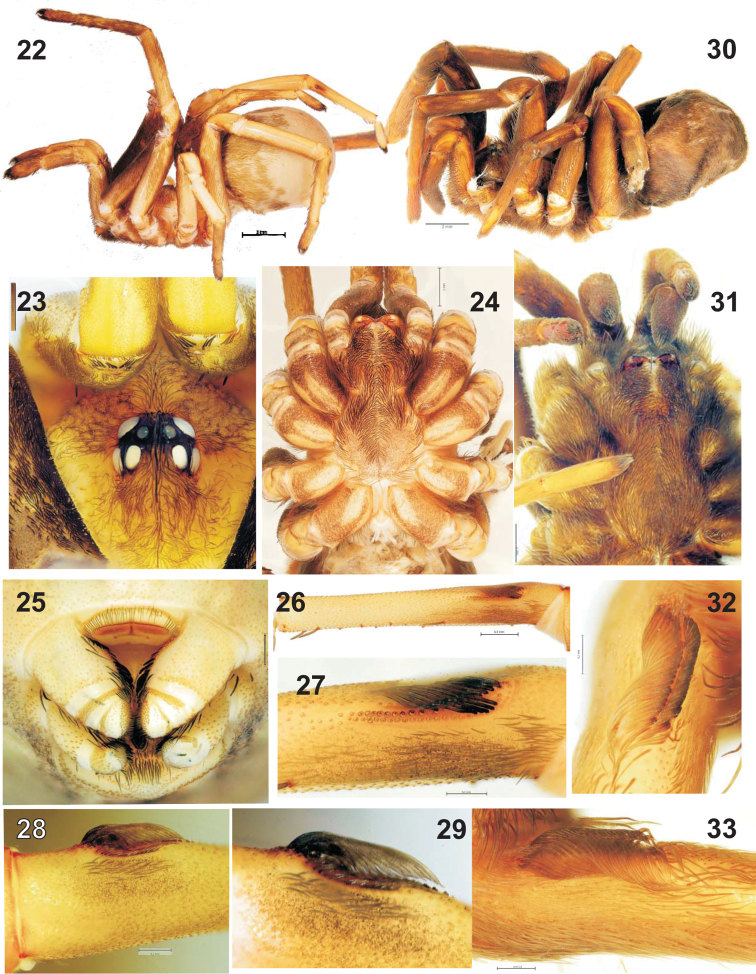
Habitus and somatic characters of two *Sahastata* species: *Sahastata sinuspersica* sp. n. (**22–29**) and *Sahastata nigra* (**30–33**). **22**, **30** habitus, lateral **23** eye group, dorsal **24**, **32** prosoma, ventral **25** spinnerets and cribellum, ventral **26**–**29**, **32**–**33** calamistrum.

Total length 12.0. Carapace 5.0 long, 4.0 wide; sternum 2.5 long, 2.25 wide. Habitus and pattern as in Figs 22–24. Palp: 2.75 + 1.45 + 1.65 + 1.6. Femur I 5.25 (other segments missing). Leg II: 4.2 + 1.7 + 3.25 + 3.35 + 1.6. Cribellar figs elongate, much longer than high (Fig. 25), calamistrum with three rows of hairs (Figs 26–29).

**Figures 34–41.**
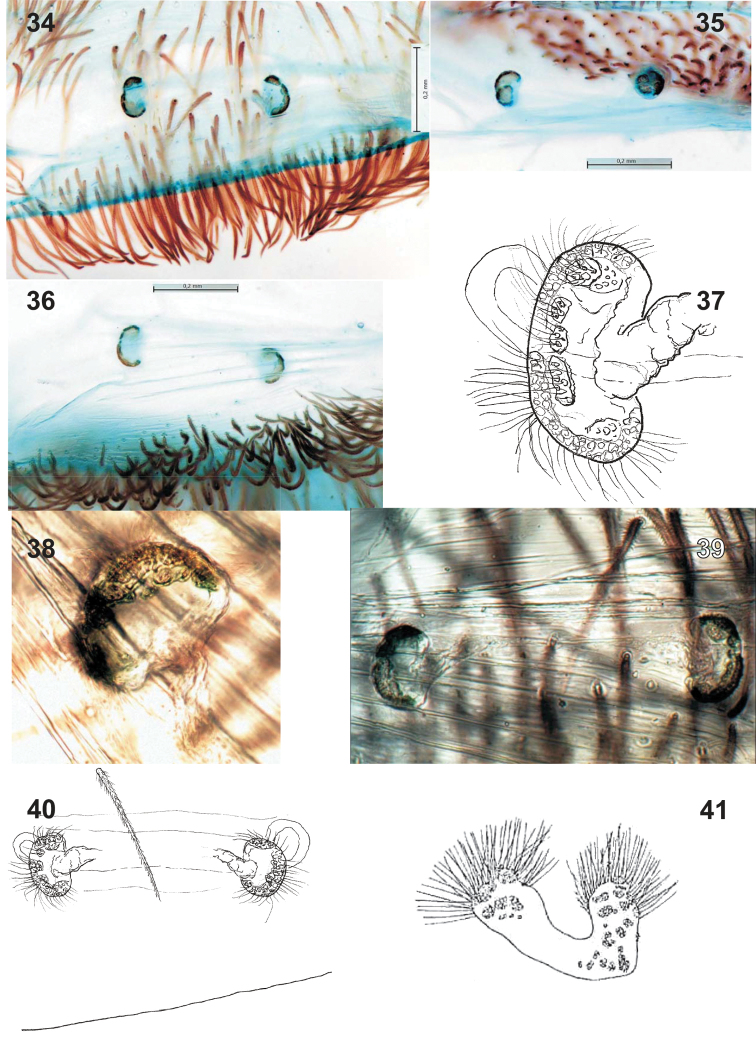
Vulvae of two *Sahastata* species: *Sahastata sinuspersica* sp. n. (**34–40**) and *Sahastata nigra* (**41**, after Benoit 1968). **34**, **39–40** dorsal **35** subapical **36** caudal **37** receptacle, subapical **38**, **41** dorsal.

Vulva as in Figs 34–40; receptacles with one distinct head standing on a membranous and goffered stalk; in apical view the receptacle appears to have two heads; head covered with isolated patches of glands; receptacles separated by about 3 diameters.

##### Distribution.

The new species is known only from the type locality in Bandar Abbas and Hormuz Island.

## Supplementary Material

XML Treatment for Anemesia

XML Treatment for Anemesia koponeni

XML Treatment for Raveniola

XML Treatment for Raveniola mazandaranica

XML Treatment for Sahastata

XML Treatment for Sahastata sinuspersica
